# Characterisation of Walker 256 breast carcinoma cells from two tumour cell banks as assessed using two models of secondary brain tumours

**DOI:** 10.1186/1475-2867-13-5

**Published:** 2013-02-01

**Authors:** Kate M Lewis, Elizabeth Harford-Wright, Robert Vink, Mounir N Ghabriel

**Affiliations:** 1 Adelaide Centre for Neuroscience Research, School of Medical Sciences, The University of Adelaide, Adelaide, South Australia, Australia

**Keywords:** Walker 256 cells, Brain metastases, Breast cancer, Animal models, Blood–brain barrier, Tumour banks, Glial reaction, Tumorigenicity

## Abstract

**Background:**

Metastatic brain tumours are a common end stage of breast cancer progression, with significant associated morbidity and high mortality. Walker 256 is a rat breast carcinoma cell line syngeneic to Wistar rats and commonly used to induce secondary brain tumours. Previously there has been the assumption that the same cancer cell line from different cell banks behave in a similar manner, although recent studies have suggested that cell lines may change their characteristics over time *in vitro*.

**Methods:**

In this study internal carotid artery injection and direct cerebral inoculation models of secondary brain tumours were used to determine the tumorigenicity of Walker 256 cells obtained from two cell banks, the American Type Culture Collection (ATCC), and the Cell Resource Centre for Medical Research at Tohoku University (CRCTU).

**Results:**

Tumour incidence and volume, plus immunoreactivity to albumin, IBA1 and GFAP, were used as indicators of tumorigenicity and tumour interaction with the host brain microenvironment. CRCTU Walker 256 cells showed greater incidence, larger tumour volume, pronounced blood–brain barrier disruption and prominent glial response when compared to ATCC cell line.

**Conclusions:**

These findings indicate that immortalised cancer cell lines obtained from different cell banks may have diverse characteristics and behaviour *in vivo*.

## Background

Cancer research has received much attention and funding over the past decades, reflecting its increased incidence and significance as a public health problem. Carcinogenesis is a multifaceted and complex disease process, making malignancies inherently difficult to treat, while at the same time presenting multiple pathways for investigation as management options. Novel treatments targeting these different pathways can then be assessed, although tumours in the brain have been excluded from many clinical trials due to the restrictive nature of the blood–brain barrier (BBB), often making brain metastases not accessible to novel treatments [1,2]. Metastatic brain tumours are present in 22-30% of patients diagnosed with breast cancer [3-5], therefore making animal models of brain metastases important tools to explore adequate treatment options for this aspect of the disease.

The process of brain metastases involves cells from a primary tumour entering blood vessels, avoiding death signals in the circulation, then undergoing extravasation through the BBB [6]. The BBB is a dynamic interface between the cerebral circulation and brain tissue, and acts to protect the brain microenvironment [7]. While investigating metastases, many scientists using cell culture presume that tumour cell lines will behave indefinitely in a uniform manner, although several studies have demonstrated that this is not the case. Changes exhibited with extended *in vitro* growth time, high passage number and cross contamination with other cell lines have been frequently described in the literature [8-11], particularly when cancer cell lines are obtained from sources other than reputable major cell libraries [12]. There is the assumption that well characterised cell lines available from cancer cell repositories are verified and maintained at a high standard, meaning that researchers do not need to authenticate these cell lines before commencing their experiments [13]. In the current study we report differential characteristics of the same cancer cell line obtained from two different reputable cell banks, suggesting that researchers cannot assume that cells obtained from reputable cancer cell repositories will all behave identically.

## Results

### DNA fingerprinting

The two Walker 256 cell lines from ATCC and the CRCTU were compared using DNA fingerprinting and shown to be of Sprague–Dawley rat origin without contamination by cell lines of other mammalian species. There is no existing reference DNA profile for the Walker 256 cell line, so it is impossible to authenticate the two cell populations used in this study. However, when compared to each other the ATCC and CRCTU Walker 256 cells had similar genetic profile with several markers that theses cell populations had in common, although many markers had different allele sizes (Table 1).

**Table 1:** DNA Fingerprinting

**Marker**	**CRCTU Walker 256**	**ATCC Walker 256**
**Name**	**Allele**	**Allele**
	**Size**	**Size**
1	97	97, 105
2	128, 130	128
3	163, 165, 183	163
4	238, 252	238, 252
42	155, 157	145
8	235, 239	235, 237
13	121, 123	121
15	248	245
16	251, 253, 273	243, 261
19	177, 179	177
24	250	259
26	159, 162, 171	152, 154
30	183, 187	187, 194
34	187, 189	187, 189
35	203, 209	203
36	224, 228	222
55	198, 206, 208	206
59	150	146
61	111	128
62	152, 155	167, 177
67	166	162
69	137, 148	139, 150
70	159, 170, 180	176
73	194, 200, 211	217
75	138, 144, 149	144, 184
78	153	151
79	172, 178, 184	172
81	130, 132	130
90	168, 172	162, 175
91	215, 222	211, 215
96	211, 213	211

### Cell morphology

**Figure 1:**
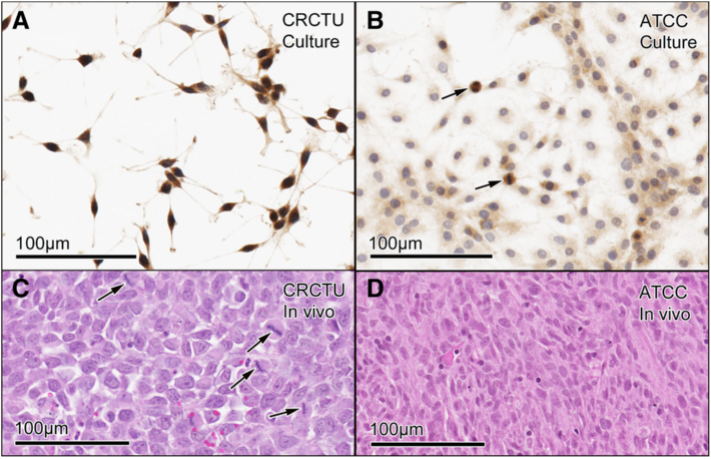
**Tumour cell morphology *in vitro *and *in vivo*. **(**A**) CRCTU Walker 256 cells in culture stained for cytokeratin 18, showing spicular appearance with deeply-stained nuclei. (**B**) ATCC Walker 256 cell in culture stained for cytokeratin 18 showing flattened cells with a large cytoplasmic component and lightly stained nuclei and mitotic figures (arrows). (**C**) CRCTU Walker 256 tumour cell *in vivo*, 9 days following internal carotid artery injection, stained with haematoxylin and eosin showing large nuclei and scanty cytoplasm with many mitotic figures (arrows). (**D**) ATCC Walker 256 tumour cell *in vivo*, 10 weeks following internal carotid artery injection, showing smaller nuclei and abundant elongated cytoplasmic component, giving the section an eosinophilic appearance.

In cell culture, both the Walker 256 cell populations received from the CRCTU and the ATCC grew very effectively, although with very different cell morphology. The cells from the CRCTU were small and spicular in appearance with deeply stained nuclei (Figure 1A), whereas the cytoplasm of the ATCC cells was abundant and the cells had a larger, flatter appearance with open face lighter stained nuclei (Figure 1B). Nuclei of the two cell populations were comparable in size (Figure 1A and B). Both the CRCTU and ATCC Walker 256 cell populations stained positively for cytokeratin 18, a marker of breast cancer cells (Figure 1A and B).

When the CRCTU and ATCC Walker 256 breast carcinoma cells were delivered to the brain through internal carotid artery injection, the resultant tumours also showed different cell morphology. The tumours from CRCTU Walker 256 cells that grew 9 days following internal carotid artery injection showed cells with large nuclei and scanty cytoplasm (Figure 1C). In contrast, the single tumour that grew 10 weeks following internal carotid artery injection of ATCC Walker 256 cells showed spindle-shaped cells with smaller nuclei and a larger cytoplasmic component (Figure 1D). Furthermore, very few mitotic figures in the ATCC tumour were seen, whereas CRCTU tumours exhibited several cells undergoing replication in any field of view (Figure 1C).

### Tumorigenicity

The CRCTU Walker 256 cells grew much more aggressively *in vivo* than the ATCC population as indicated by the earlier sacrifice time required for the CRCTU injected animals in both models. Following internal carotid artery injection, only one animal of 9 injected with ATCC cells developed a metastatic brain tumour at the 10 week time point, whereas 8 out of the 9 animals injected with the CRCTU cells showed tumours at the late time point of 9 days (Table 2). Furthermore, the CRCTU internal carotid artery injected animals also showed metastatic brain tumours in one out of the 5 animals killed at the intermediate time point of 6 days following surgery (Table 2). Neither the CRCTU nor the ATCC Walker 256 injected animals showed any evidence of tumour growth at the early time point of 24 hours post internal carotid artery injection (Table 2). The single tumour that resulted from carotid injection with ATCC Walker 256 cells was located in the striatum. In contrast, the tumour masses in the CRCTU Walker 256 injected animals were predominantly found in the lateral ventricles.

**Table 2:** Tumour incidence

**Internal carotid artery i njection**	**CRCTU**		**ATCC**	
Early time point (n=5)	24 hours	0% (0/5)	24 hours	0% (0/5)
Intermediate time point (n=5)	6 days	20% (1/5)	4 weeks	0% (0/5)
Late time point (n=9)	9 days	89% (8/9)	10 weeks	11% (1/9)
**Direct inoculation**	**CRCTU**		**ATCC**	
(n=6)	7 days	100% (6/6)	4 weeks	0% (0/6)

Tumour incidence in animals injected via the internal carotid artery or directly inoculated into the brain with Walker 256 breast tumour cells obtained from the Cell Resource Centre for Medical Research at Tohoku University (CRCTU) or the American Type Culture Collection (ATCC).

**Figure 2:**
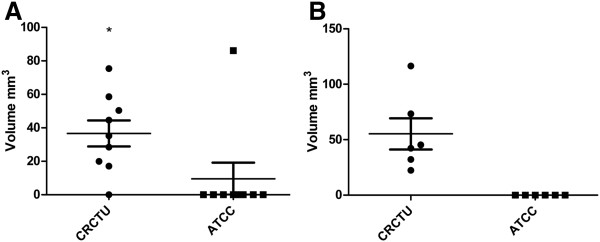
**Tumour volume. **(**A**) Tumour volume following internal carotid artery injection with CRCTU and ATCC Walker 256 rat carcinoma cells at 9 days and 10 weeks, respectively, following surgery showing. Only a single ATCC Walker 256 inoculated animal exhibited tumour growth (*p<0.05). (**B**) Tumour volume following direct inoculation of CRCTU and ATCC cells into the right striatum 7 days and 4 weeks respectively following surgery, only CRCTU Walker 256 inoculated animals grew metastatic brain tumours of substantial volume.

Similar to the internal carotid artery injection model, the CRCTU cells were more effective in producing metastatic brain tumours when inoculated directly into the brain, compared to the ATCC cells (Table 2). Direct inoculation of CRCTU tumour cells into the striatum resulted in development of large neoplastic masses in the brain tissue of 100% of the animals, whereas none of the ATCC Walker 256 inoculated animals showed any evidence of tumour growth (Table 2). Comparison of the two models used in this study revealed that direct injection of CRCTU Walker 256 cells into the brain resulted in larger and more consistent location of tumour growth in the striatum with a mean volume of 55.28 mm^3^, compared with an average tumour volume of 36.61mm^3^ following internal carotid artery injection of the same CRCTU Walker 256 cells (Figure 2A and B).

**Figure 3:**
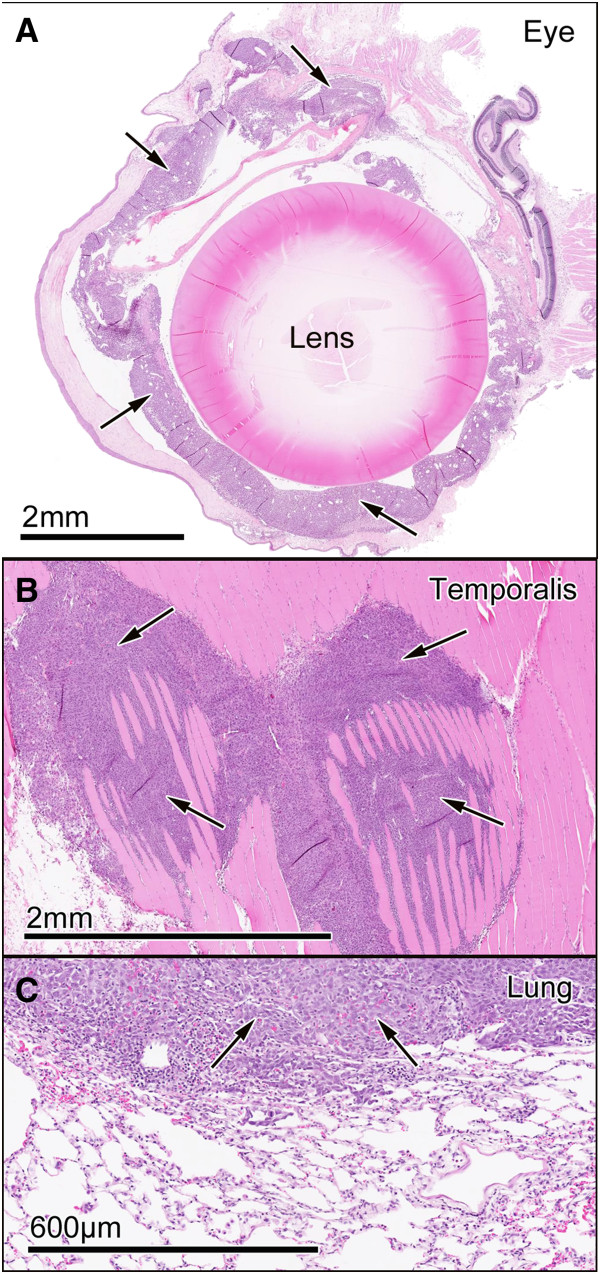
**Extracranial tumour growth. **(**A**) CRCTU Walker 256 tumour growth (arrows) in the eye 9 days following internal carotid artery injection stained with haematoxylin and eosin. (**B**) CRCTU Walker 256 tumour growth (arrows) invading the temporalis muscle 9 days post internal carotid artery injection, stained with haematoxylin and eosin. (**C**) Lung section stained with haematoxylin and eosin, showing a large tumour mass (arrows) 9 days following CRCTU Walker 256 cells carotid injection.

All the animals that developed metastatic brain tumours in the 9 day CRCTU group showed a concurrent growth of a tumour in the right eye (Figure 3A). Also 44.4% of these animals had small tumour nodules in the right temporalis muscle, and 33.3% developed lung tumours (Figure 3B and C). None of these features were seen in the animals injected with ATCC Walker 256 cells, or with animals inoculated directly into the striatum with the CRCTU Walker 256 cells.

### Tumour interactions with the BBB

In the present study, albumin was used as an endogenous marker of BBB permeability, given that serum albumin is confined to blood vessels under normal conditions. However, when the BBB is compromised, albumin leaks out of the blood vessels into the surrounding neuropil. Separate control groups for different time points were required for the direct inoculation model, due to the invasive nature of the surgery. In contrast, injection of culture medium into the internal carotid artery did not cause variation of blood–brain barrier disruption over time, and only one control group was used for all time points.

**Figure 4:**
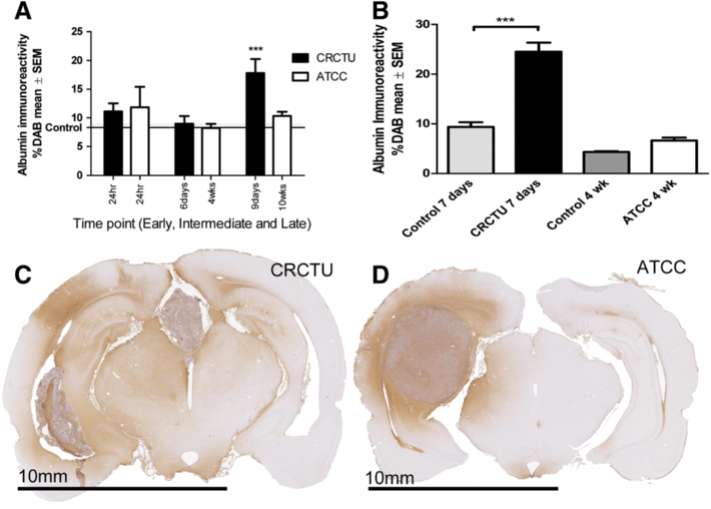
**Albumin immunoreactivity. **(**A**) Graph showing %DAB in albumin immunostained coronal sections of the brain at early, intermediate and late time points following internal carotid artery injection of CRCTU and ATCC Walker 256 tumour cells when compared to culture medium control brains; ***p<0.001. (**B**) Graph showing %DAB in albumin immunostained brain coronal sections 7 days and 4 weeks following CRCTU and ATCC Walker 256 tumour inoculation respectively, compared to culture medium control brains; ***p<0.001 (**C**) Coronal brain section stained for albumin 9 days following internal carotid artery injection of CRCTU Walker 256 breast carcinoma cells showing widespread immunoreactivity mainly in the right hemisphere. (**D**) Albumin immunostained brain coronal section 10 weeks post internal carotid artery injection with ATCC Walker 256 breast carcinoma cells showing peritumoral immunoreactivity.

Neither CRCTU nor ATCC Walker 256 tumour injection into the internal carotid artery caused a significant increase in albumin immunoreactivity 24 h following surgery, when compared to the culture medium control group (Figure 4A). A similar pattern of immunoreactivity was evident at the intermediate time point following internal carotid artery injection of Walker 256 cells from both cell banks (Figure 4A). In contrast, by 9 days following CRCTU Walker 256 internal carotid artery injection there was a significant increase in albumin immunoreactivity in the brain coronal sections when compared to the culture medium control group (p<0.001; Figure 4A). Similarly, only CRCTU Walker 256 inoculated and not ATCC Walker 256 inoculated brains showed a significant increase in albumin immunoreactivity following direct injection of tumour cells into the striatum when compared to the respective culture medium control group (p<0.001; Figure 4B).

Widespread albumin immunoreactivity was evident throughout the brains in animals that grew tumours after receiving CRCTU Walker 256 cells by the internal carotid artery injection or via direct inoculation into the brain (Figure 4C). This indicates that the tumours that result from CRCTU cell had widespread effects on BBB permeability. In contrast, the increase in BBB permeability was more concentrated in the immediate vicinity of the single tumour that formed after ATCC Walker 256 tumour injection into the internal carotid artery (Figure 4D).

### Brain microenvironment

**Figure 5:**
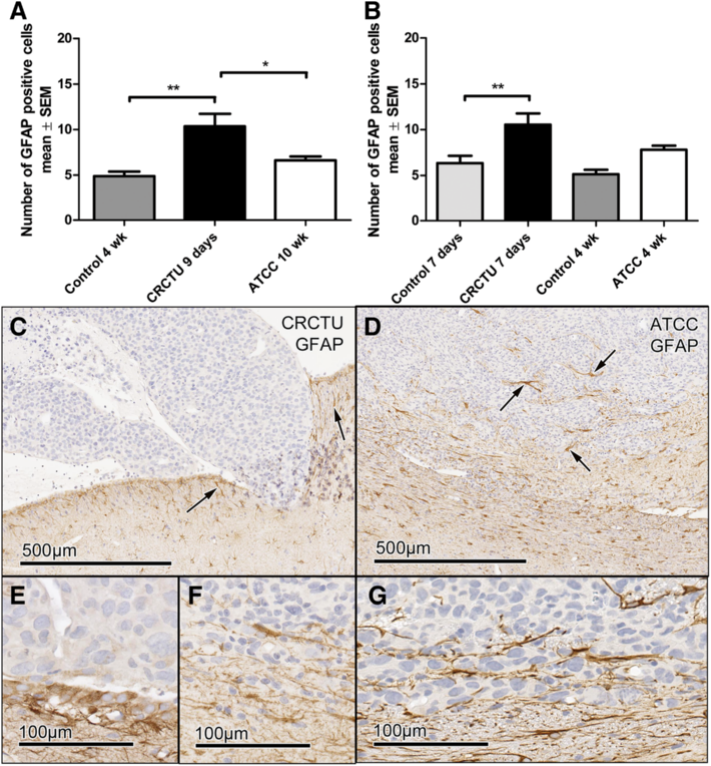
**GFAP immunoreactivity. **(**A**) Graph showing the average number of GFAP positive cells in 4 areas of the cortex (0.0678 mm^2^) in animals injected with culture medium, or injected with CRCTU or ATCC Walker 256 cells into the internal carotid artery; **p<0.01; *p<0.05. (**B**) Graph showing the average number of GFAP positive cells in 4 areas of the striatum (0.0678 mm^2^) following direct inoculation of culture medium, CRCTU or ATCC Walker 256 cells into the brain; **p<0.01. (**C**) GFAP immunostained section 9 days following CRCTU Walker 256 internal carotid artery injection, showing absence of staining within the tumour, but showing GFAP labelled cell in peritumoral area (arrows). (**D**) Brain section stained for GFAP 10 weeks following carotid ATCC Walker 256 tumour cell injection. Labelled astrocytes (arrows) are seen in the peritumoral area and in the periphery of the tumour mass between the tumour cells. (**E**) High magnification of GFAP immunostained section 9 days following CRCTU Walker 256 internal carotid artery injection. (**F**) High magnification of GFAP immunostained section 10 weeks following ATCC Walker 256 internal carotid artery injection. (**G**) GFAP immunostained section 7 days following direct inoculation of CRCTU Walker 256 inoculation showing substantial peritumoral immunoreactivity.

**Figure 6:**
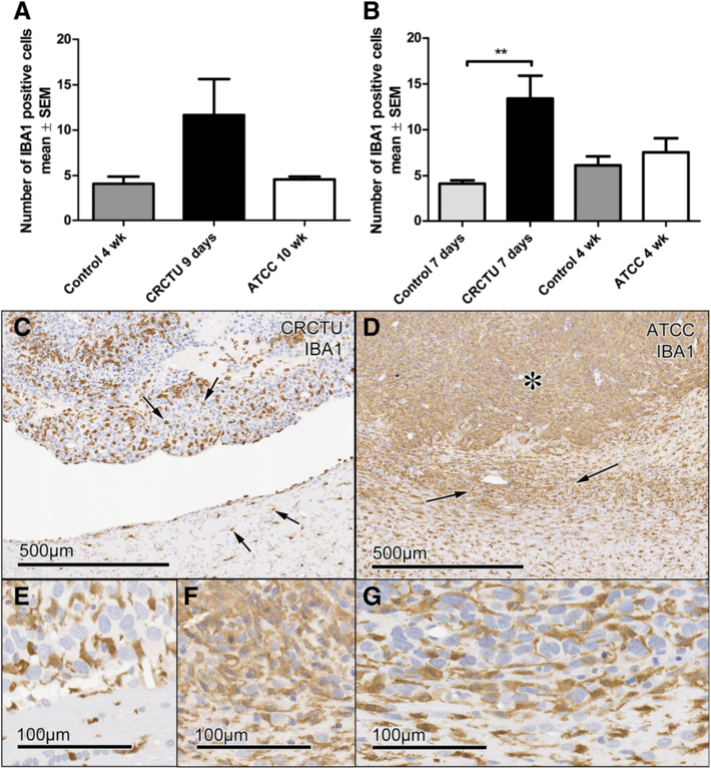
**IBA1 immunoreactivity. **(**A**) The average number of IBA1 positive cells /0.0678 mm^2 ^of the cortex in animals injected with culture medium, CRCTU or ATCC Walker 256 breast carcinoma cells into the internal carotid artery. (**B**) The average number of IBA1 positive cells /0.0678 mm^2 ^of the striatum following direct inoculation of culture medium, CRCTU or ATCC Walker 256 cells into the brain; **p<0.01. (**C**) IBA1 immunostained brain 9 days following internal carotid artery inoculation with CRCTU Walker 256 cells, arrows showing labelled cells dispersed between cancer cells and in the peritumoral area. (**D**) IBA1 immunostained brain section showing extensive labelling within the tumour mass (asterisk) and in the peritumoral area (arrows) 10 weeks following ATCC Walker 256 breast carcinoma cell injection into the internal carotid artery. (**E**) Higher magnification IBA1 immunostained section 9 days following CRCTU Walker 256 internal carotid artery injection. (**F**) Higher magnification IBA1 immunostained section 10 weeks following ATCC Walker 256 internal carotid artery injection. (**G**) IBA1 immunostained section 7 days following direct inoculation of CRCTU Walker 256 inoculation showing increased immunoreactivity in the peritumoral area along with IBA1 labelled cells within the tumour mass.

Both models of metastatic tumour induction caused changes in the brain microenvironment when CRCTU Walker 256 breast carcinoma cells were utilised (Figures 5 and 6). There was a significant increase in the number of GFAP positive cells in the cortex of animals 9 days following internal carotid artery injection of CRCTU Walker 256 cells when compared to the culture medium control group (p<0.01; Figure 5A). Correspondingly, there was a significant increase in the number of astrocytes immunostained for GFAP in the striatum surrounding the tumour mass 7 days following direct injection of CRCTU Walker 256 cells (p<0.01; Figure 5B). In contrast, ATCC Walker 256 cells administered via either the internal carotid artery injection or direct inoculation into the striatum did not significantly alter the number of GFAP labelled cells when compared to the same location in culture medium inoculated brains (Figure 5A, B).

GFAP immunoreactivity was absent within the tumour masses for the CRCTU Walker 256 internal carotid artery model, indicating the absence of astrocytes within the tumours (Figure 5C). However, the single tumour that grew 10 weeks following internal carotid artery injection of ATCC Walker 256 tumour cells showed an increase in GFAP labelled cells in the peritumoral area and some infiltrating labelled cells within the periphery of the tumour (Figure 5D). The astrocytes surrounding the tumour mass exhibited short, blunt, thickened processes, with the flattened cells creating a limiting rim (Figure 5D). The tumours that grew 9 days following internal carotid artery injection of CRCTU Walker 256 cells within the lateral ventricles had limited contact with the neuropil and lacked the GFAP positive astrocytic border that was evident around the ATCC Walker 256 tumour (Figure 5C).

Tumour cell inoculation caused an increase in the number of microglia, as indicated by IBA1 labelling, in the cortex of brains 9 days following internal carotid artery injection of CRCTU Walker 256 cells in comparison to control brains (Figure 6A). Similarly, 7 days after direct injection of CRCTU Walker 256 cells into the brain, there was a significant increase in IBA1 positive cells in the striatum surrounding the tumour mass, when compared to the same location in the culture medium control group (p<0.01; Figure 6B). However, the increase in microglia seen with CRCTU Walker 256 cell inoculation was not replicated by ATCC Walker 256 cells when injected into the internal carotid artery or inoculated directly into the striatum (Figure 6A and B).

Examination of brain sections immunolabelled for IBA1 showed a distinct pattern of staining for each Walker 256 cell type. The CRCTU tumours showed sparse but specific discrete labelling of infiltration by microglia (Figure 6C). In contrast, the ATCC tumour showed more widespread ill-defined labelling throughout the tumour mass (Figure 6D). Furthermore, there was a halo of IBA1 labelled cells surrounding the tumour mass following internal carotid artery inoculation of ATCC Walker 256 cells, a feature that was not present surrounding the CRCTU Walker 256 induced tumours (Figure 6C and D).

## Discussion

In the current study, Walker 256 cells obtained from the CRCTU had potent tumorigenic properties when compared to the ATCC Walker 256 breast carcinoma cells. Evidence of this includes the substantially increased incidence of tumour growth and tumour volume after CRCTU Walker 256 inoculation in the two tumour models used in this study, as well as the fact that only CRCTU Walker 256 internal carotid artery injected animals developed tumours in the eye, temporalis muscle and lung. It has been shown in previous studies that different tumour cell lines cloned from the same neoplasm may have different tumorigenic properties when implanted *in vivo*[14,15]. However, cell lines developed from a single mouse mammary tumour that showed differing culture morphology and growth characteristics *in vitro*, resulted in tumours that displayed similar histology to each other and comparable tumorigenicity when injected into syngeneic hosts [16].

Despite the fact that both populations of Walker 256 breast carcinoma cells were obtained from reputable tumour cell banks that described the Walker 256 cell line as tumorigenic in Wistar rats, there was considerable variability in their genetic profile and subsequently growth behaviour *in vivo* and morphology *in vitro*. This was despite the fact that both Walker 256 cell lines used in this study were shown to be of rat origin with no evidence of contamination by other mammalian cell lines. ATCC has been instrumental in the push to develop a standard method of cell line verification involving short tandem repeat profiling along with the development of a database of short tandem repeat profiles for commonly used cell lines [17,18].

Control of cancer cell tumorigenicity has been extensively studied, predominantly in relation to genetic control of cancer growth *in vivo*. For example, p75 has been linked to reduced neuroblastoma tumorigenicity [19]. However, characteristics of tumour cells in culture have also been investigated, with shorter doubling time, reduced monolayer density, poor motility and lower incidence of focus formation *in vitro* linked to decreased tumorigenicity of cell lines when used *in vivo*[20,21], although these experiments were generally comparing different cell lines. In contrast, the current study aimed to determine the differences between the same cell line obtained from two different sources.

The CRCTU Walker 256 breast carcinoma cells, found to be more tumorigenic than their ATCC counterparts, showed darker nuclear staining and increased nucleus to cytoplasm ratio when compared to the flatter more eosinophilic ATCC Walker 256 cells. There have been few previous studies to determine the relationship between cell morphology and cancer cell tumorigenicity. Further investigation is required to determine if the characteristics observed in this experiment are related to the tumorigenicity of the cells described. Furthermore, previous studies have suggested that behaviour of cancer cell lines *in vitro* is poorly correlated with tumorigenicity *in vivo*[21]. Despite this, in the current study morphological features seen *in vitro* for Walker 256 cells from both the CRCTU and ATCC were closely associated with the morphology evident *in vivo*.

There are many plausible explanations for the differential characteristics evident for CRCTU and ATCC Walker 256 breast carcinoma cells in this study. It is possible that variations in storage methods, extended culture times and high passage number may have contributed to the differences seen in the same cell line obtained from the CRCTU and the ATCC. Immortalised tumour cell lines evolve over time in animal models where malignancies are induced by inoculation with a homogenous population of tumour cells [22]. Conversely, human neoplastic tissue is not a uniform entity. Within a tumour mass, there exist various heterogeneous subpopulations of tumour cells with different metastatic potential and diverse propensity to metastasise to various organs [23,24].

Tumour cells harvested from a neoplasm *in vivo* have been known to develop characteristics over time *in vitro* that are distinct from those evident in the original cancerous tissue [14]. The proposed reason for this phenotypic change is that more aggressive or mitotic properties are favoured by clonal selection *in vitro*, with highly metastatic varieties more phenotypically stable [25,26]. Long term passage of Walker 256 cells has previously been shown to alter chemotactic behaviour *in vitro*[27].

Walker 256 carcinoma is rat mammary tumour cell line that originally occurred spontaneously in a pregnant albino Sprague-Dawley rat [28]. The Walker 256 cell line has been used previously to establish experimental brain metastases through an internal carotid artery injection and direct implantation into the cerebral cortex [29-33].

Tumour growth evident following both inoculation methods of CRCTU Walker 256 cells showed larger tumour volume in a shorter period of time, when compared to previous experiments described in the literature using the Walker 256 cell line, although the incidence was comparable [34-37]. In contrast, the ATCC Walker 256 cells showed a much lower incidence and longer incubation period required to form only a single tumour when compared to these previous studies. Therefore, neither the CRCTU, nor the ATCC Walker 256 breast carcinoma cells behaved exactly as previous studies have described, although the CRCTU population were more analogous to the literature.

Despite the consistency of the direct injection model of tumour induction, the ATCC Walker 256 cells did not grow any tumours through the use of this method. Thus the extravasation process through the BBB is not the limiting factor for ATCC Walker 256 tumour growth in the brain. Furthermore, 11% of animals grew metastatic brain tumours 10 weeks following ATCC Walker 256 inoculation into the internal carotid artery, meaning that at least some of the tumour cells were able to complete the extravasation process.

The CRCTU Walker 256 inoculated animals for both the internal carotid artery and the direct inoculation model showed a significant increase in albumin immunoreactivity when compared to the culture medium group. It is likely that albumin immunoreactivity was increased in response to the substantial tumour growth evident in the CRCTU Walker 256 tumour inoculated groups and subsequent increased BBB permeability. It is well accepted in the literature that blood vessels within brain metastases of breast cancer are more permeable than BBB microvessels, as they are characteristic of the breast tissue origin of the tumour cells causing substantial cerebral oedema [29,38-41]. Furthermore, it has been postulated that the permeability of blood vessels surrounding brain metastases is also increased, which may explain the widespread albumin immunoreactivity evident 9 days following CRCTU tumour injection into the internal carotid artery.

The ATCC tumour cell inoculated animals only grew one tumour in either model of metastatic brain tumour induction, which was not sufficient to cause a significant difference in albumin immunoreactivity from vehicle level and thus did not increase the permeability of the BBB. This shows that the presence of tumour cells with low tumorigenicity in the brain microcirculation do not cause an inflammatory reaction disrupts the normal function of the BBB. Furthermore, ATCC Walker 256 localisation in the neuropil of the striatum did not cause long term damage to the brain sufficient to increase the permeability of the BBB 4 weeks following direct injection.

A rim of reactive glial cells is often evident surrounding metastatic brain tumours in human surgical tissue [42], as was also apparent surrounding tumours grown in this study. The pattern of glial cell reaction was different surrounding CRCTU and ATCC Walker 256 tumours that grew following internal carotid artery inoculation. The location of CRCTU tumours within the lateral ventricles may be the cause of these differences, as the mass is in less direct contact with the neuropil. In contrast, the single tumour that grew 10 weeks following internal carotid artery inoculation of ATCC Walker 256 cells, showed much more extensive microglial infiltration along with increased microglia and astrocytes surrounding the tumour. A proposed function of this glial halo is to act as a barrier to the flow of oedematous fluid [43]. Astrocytes and microglia may proliferate and become activated in response to contact with serum proteins, such as albumin which is present in oedematous fluid that accumulates around the tumour [33,44]. However it is also possible that in the direct inoculation model the glial reaction could be caused in part by reaction to needle track injury, particularly for the animals that were euthanized 7 days following CRCTU Walker 256 inoculation.

The low tumorigenicity of ATCC Walker 256 cells may be the reason that these cells did not show the same influence on the brain microenvironment as CRCTU Walker 256 growth. This is demonstrated by the significant increase in IBA1 and GFAP labelled cells following both internal carotid artery and direct injection of CRCTU Walker 256 tumour cells when compared to the culture medium injected groups. However this phenomenon was not evident following ATCC Walker 256 tumour inoculation for either model used in this study. Thus, the presence of low tumorigenicity cancer cells in the brain microcirculation or the neuropil, did not show significant interaction with the host microenvironment.

## Conclusions

In conclusion, this study has demonstrated that the Walker 256 tumour cells obtained from two reputable sources have different tumorigenicity, growth characteristics and interactions with the host brain. Such variability should be considered when comparing studies using the same cell line obtained from different sources.

## Methods

### Cell culture

Walker 256 breast tumour cells (rat) were obtained from two cell banks, the American Type Culture Collection (ATCC), and the Cell Resource Centre for Medical Research at Tohoku University (CRCTU). The Walker 256 cells obtained from the ATCC were reported to be passage number 290. However, the CRCTU did not provide details of passage number for the Walker 256 cells. These two cell populations were cultured for a maximum of 30 additional passages, in the same incubator. Both the Walker 256 cell populations were cultured according to the instructions from the respective cell bank. Briefly, Walker 256 cells from ATCC were cultured in growth medium consisting of Sigma 199 M4530 culture medium containing 5% sterile normal horse serum and 1 mL of penicillin and streptomycin (Sigma 10,000 units of penicillin and 10 mg of streptomycin/mL) for each 100 mL volume, while Walker 256 cells from the CRCTU were cultured in growth medium made up of Sigma RPMI-1640 culture medium containing 10% sterile foetal bovine serum and 1 mL of penicillin and streptomycin (Sigma 10,000 units penicillin and 10 mg of streptomycin/mL) for each 100 mL volume.

Culture flasks of 150 cm^2^ were used to grow the cells and once >90% confluence was reached, the cells were detached by the addition of 3.5 mL of 1% trypsin (Sigma) or 3.5 mL of 0.02% EDTA for ATCC and CRCTU Walker 256 cells, respectively. The cells were spun down in a centrifuge (5 minutes at 1500 RPM) and then resuspended in serum free culture medium. The number of cells was calculated using a haemocytometer and then diluted, so that there was between 10^5^ and 10^6^ cells in every 0.2 mL of cell suspension for internal carotid artery injection, or the same number of cells in 8 μL for direct inoculation into the brain.

### Animals

The experimental procedures described throughout this project were performed within the National Health and Medical Research Council (NHMRC) guidelines and were approved by both the University of Adelaide and Institute of Medical and Veterinary Science Animal Ethics Committees. All experiments complied with the EC Directive 86/609/EEC for animal experiments. Male Wistar rats weighing 250-350 g were group housed in the IMVS Animal Facility and were supplied with a diet of rodent pellets and water ad libitum. Animals were randomly selected for either the internal carotid injection procedure or the direct inoculation procedure and then were further divided into culture medium only control group, Walker 256 tumour CRCTU group and Walker 256 tumour ATCC group.

### DNA fingerprinting of cell lines

The Walker 256 cell lines obtained from the CRCTU and ATCC were submitted to IDEXX RADIL for DNA fingerprinting, using 31 short tandem repeat markers that are rat specific in order to establish the genetic profile of the two cell populations. Cell samples were also tested for cross species contamination.

### Internal carotid artery injection

Animals allocated to the internal carotid injection procedure were sacrificed at 24 h (early, n=5), 6 days (intermediate, n=5) and 9 days (late, n=9) for the CRCTU Walker 256 cells, and at 24 h (early, n=5), 4 weeks (intermediate, n=5) and 10 weeks (late, n=9) for the ATCC Walker 256 cells. The selected late time points were determined after a pilot study of tumour burden and animal weight loss for both cell lines. The method for internal carotid artery injection of tumour cells to induce metastatic brain tumour growth has been previously described in detail [45]. Briefly, under 2% isoflurane inhalation anaesthesia via endotracheal tube, a longitudinal skin incision was made to expose the carotid bifurcation. The ophthalmic artery, superior thyroid artery and pterygopalatine artery were occluded to specifically deliver tumour cells to the brain. The external carotid artery was sacrificed, forming a surgical stump to provide an access point for cannulation of the internal carotid artery for injection of 0.2 mL of culture medium or tumour cell suspension, following temporary occlusion of the common carotid artery. Once the cannula was removed and a suture tied around the external carotid stump, blood flow through the common carotid artery was re-established and the wound sutured.

### Direct inoculation

Animals that received direct intraparenchymal inoculation were sacrificed at 7 days and 4 weeks for the CRCTU and ATCC Walker 256 cells, respectively (n=6/group). Direct stereotaxic inoculation of tumour cells into the right striatum for induction of metastatic brain tumour has been previously described in detail [46]. Briefly, animals were anaesthetised using 3% isoflurane inhalation anaesthesia via a nose cone, placed in a stereotactic frame and a midline scalp incision made to expose the skull. A 0.7 mm burr hole was performed at stereotaxic coordinates: anterior 0.5 mm, lateral 3 mm to the right relative to the bregma. A 30 gauge Hamilton syringe was inserted and lowered stereotaxically 5 mm and 8 μL of culture medium or tumour cell suspension injected directly into the brain tissue over 10 minutes. 5 minutes following inoculation, the needle was removed, the hole was sealed with bone wax and the wound sutured.

### Tumour volume

For histological analysis, animals were transcardially perfused with 10% formalin under terminal anaesthesia induced by intraperitoneal administration of pentobarbitone sodium (60 mg/kg). Brains were embedded in paraffin wax and sequential 5 μm coronal sections were cut from blocks 2mm thick in a rostro-caudal direction, to be used for haematoxylin and eosin staining and immunohistochemistry. The haematoxylin and eosin stained slides were scanned using a Nanozoomer (Hamamatsu, Hamamatsu City, Japan) and images used to calculate tumor volume. This was performed by determining the area of tumour in each section using the NDP viewer programme and multiplying the area by the distance between sections as previously described [47].

### Immunostaining

Slides from each model were stained for albumin (ICN Pharmaceuticals, 1:20,000), glial fibrillary acidic protein (GFAP, Dako, 1:40,000) and ionized calcium binding adaptor molecule 1 (IBA1, Dako, 1:50,000). Tumour cells were also grown on cover-slips *in vitro* to be immunostained for cytokeratin18 (Gene Tex, 1:3,000). Immunohistochemistry was performed using the standard streptavidin procedure with 3,3^′^-diaminobenzidine (DAB) for visualization and haematoxylin counterstaining. Slides were scanned using the Nanozoomer. Albumin immunostaining, expressed as the weighted %DAB in each coronal section, was estimated using colour deconvolution techniques, as described previously [48,49]. For GFAP and IBA1 immunoreactivtiy, 4 fields of view were taken from the cortex and striatum for the internal carotid artery injection model and the direct inoculation model. The immunolabelled cells in these images were counted and the mean number calculated for all images from each brain.

### Statistical analysis

Results were expressed as mean±SEM and an unpaired t test (for two groups) or a one-way analysis of variance followed by a Bonferroni post test (for more than two groups) performed. Values of p<0.05 were designated as significant.

## Abbreviations

(BBB): blood–brain barrier; (ATCC): American Type Culture Collection; (CRCTU): Cell Resource Centre for Medical Research at Tohoku University; (DAB): 3,3′-diaminobenzidine; (GFAP): glial fibrillary acidic protein; (IBA1): ionized calcium binding adaptor molecule 1.

## Competing interests

The authors declare that they have no actual or potential conflict of interest including any financial, personal or other relationships with other people or organizations that could inappropriately influence, or be perceived to influence, our work.

## Authors’ contributions

We declare that all the authors have approved the submission of this article. We declare that all authors have contributed to scientific work and writing and editing of this article, KML as part of her PhD study, conducted the practical work and writing of the paper; EHW contributed to the discussion and critical reading of the paper; RV co-supervisor of the study, discussion throughout and critical reading and editing of the article; MNG, Principal supervisor of the study, discussion throughout, critical reading and editing the article and final submission.
